# Physical activity and lung function—Cause or consequence?

**DOI:** 10.1371/journal.pone.0237769

**Published:** 2020-08-20

**Authors:** Annabelle Bédard, Anne-Elie Carsin, Elaine Fuertes, Simone Accordini, Shyamali C. Dharmage, Vanessa Garcia-Larsen, Joachim Heinrich, Christer Janson, Ane Johannessen, Bénédicte Leynaert, José Luis Sánchez-Ramos, Gabriela P. Peralta, Isabelle Pin, Giulia Squillacioti, Joost Weyler, Deborah Jarvis, Judith Garcia-Aymerich

**Affiliations:** 1 ISGlobal, Barcelona, Spain; 2 Universitat Pompeu Fabra (UPF), Barcelona, Spain; 3 CIBER Epidemiologia y Salud Pública (CIBERESP), Barcelona, Spain; 4 National Heart and Lung Institute, Imperial College London, London, United Kingdom; 5 Unit of Epidemiology and Medical Statistics, Department of Diagnostics and Public Health, University of Verona, Verona, Italy; 6 Allergy and Lung Health Unit, School of Population and Global Health, University of Melbourne, Melbourne, Australia; 7 Program in Human Nutrition, Department of International Health, Johns Hopkins Bloomberg School of Public Health, Baltimore, Maryland, United States of America; 8 Institute and Outpatient Clinic for Occupational, Social and Environmental Medicine, University Hospital Munich, Ludwig Maximilians University Munich, Munich, Germany; 9 Department of Medical Sciences, Respiratory, Allergy and Sleep Research, Uppsala University, Uppsala, Sweden; 10 Centre for International Health, Department of Global Public Health and Primary Care, University of Bergen, Bergen, Norway; 11 Department of Occupational Medicine, Haukeland University Hospital, Bergen, Norway; 12 Inserm, UMR 1152, Pathophysiology and Epidemiology of Respiratory Diseases, Paris, France; 13 UMR 1152, University Paris Diderot, Paris, France; 14 Pneumology Service, Juan Ramón Jiménez Hospital, Huelva, Spain; 15 CHU Grenoble Alpes, Department of Pediatrics, Grenoble, France; 16 INSERM, Institut for Advanced Biosciences, Grenoble, France; 17 University Grenoble Alpes, Grenoble, France; 18 Department of Public Health and Pediatrics, University of Turin, Turin, Italy; 19 Department of Epidemiology and Social Medicine, University of Antwerp, Antwerp, Belgium; 20 MRC-PHE Centre for Environment and Health, Imperial College London, London, United Kingdom; Cincinnati Children's Hospital Medical Center, UNITED STATES

## Abstract

Concerns exist that the positive association of physical activity with better lung function, which has been suggested in previous longitudinal studies in smokers, is due to reverse causation. To investigate this, we applied structural equation modeling (SEM), an exploratory approach, and marginal structural modeling (MSM), an approach from the causal inference framework that corrects for reverse causation and time-dependent confounding and estimates causal effects, on data from participants in the European Community Respiratory Health Survey (ECRHS, a multicentre European cohort study initiated in 1991–1993 with ECRHS I, and with two follow-ups: ECRHS II in 1999–2003, and ECRHS III in 2010–2014). 753 subjects who reported current smoking at ECRHS II, with repeated data on lung function at ECRHS I, II and III, physical activity at ECRHS II and III, and potential confounders at ECRHS I and II, were included in the analyses. SEM showed positive associations between physical activity and lung function in both directions. MSM suggested a protective *causal* effect of physical activity on lung function (overall difference in mean β (95% CI), comparing active versus non-active individuals: 58 mL (21–95) for forced expiratory volume in one second and 83 mL (36–130) for forced vital capacity). Our results suggest bi-directional causation and support a true protective effect of physical activity on lung function in smokers, after accounting for reverse causation and time-dependent confounding.

## Background

Previous longitudinal population-based studies suggest a protective effect of physical activity on lung function levels among active smokers [1,2]. However, the potential for reverse causation remains a common criticism, even in longitudinal studies, as both lung function and physical activity vary over time, and previous lung function levels may have affected baseline physical activity levels. This is further complicated by the possibility of time-dependent confounding, which is where a time-varying confounder (e.g., weight) is affected by previous levels of the exposure (i.e. physical activity). One study reported that the role of time-dependent confounding in the association between physical activity and lung function was of negligible magnitude, but did not consider the influence of diet, which is closely related to physical activity and weight [3]. We investigated the potential role of reverse causation and time-dependent confounding on the association between physical activity and lung function among active smokers using repeated data from the European Community Respiratory Health Survey (ECRHS). We used statistical techniques that, unlike standard statistical methods, provide unbiased results in the presence of time-dependent confounding: structural equation modeling (SEM)–an exploratory approach and marginal structural modeling (MSM)–a causal approach.

## Methods

### Study population

The ECRHS multicentre cohort study collected repeated detailed information on environmental, lifestyle and respiratory health factors from adults, who were sampled in 30 centres (located in 13 European countries and Australia) and were evaluated in 1991–1993 (ECRHS I), 1999–2003 (ECRHS II) and 2010–2014 (ECRHS III). Details of the study design have already been published [4,5]. For this analysis, from the 1,578 subjects who had reported current smoking at baseline (i.e. ECRHS II in our analyses), we excluded the 488 subjects without lung function data at all ECRHS assessments, 62 subjects without physical activity data at both ECRHS II and III, and 275 subjects without dietary data at either ECRHS II or III. A total of 753 subjects from 18 centres were included in our study population (a flow-chart is provided S1 Fig in online S1 File).

### Lung function

Pre-bronchodilation forced expiratory volume in one second (FEV_1_) and forced vital capacity (FVC) were measured at each survey according to American Thoracic Society recommendations [6].

### Physical activity

At ECRHS II and III, information of usual vigorous physical activity frequency (never, ≤once a month, once a week, 2–3 times per week and ≥4 times per week) and duration per week (none, 30 minutes per week, 1 hour per week, 2–3 hours per week and ≥4 hours per week) was obtained using interviewer-administered questionnaires, at the same time as when lung function was measured. Participants were classified as either physically active if they had reported ≥2 times and ≥1 hour per week of vigorous physical activity, or non-active otherwise [2]. This “active” variable thus represents a combination of physical activity frequency and duration, and it has been shown to be associated with FEV_1_ and FVC in smokers from the ECRHS [2].

### Other relevant information

Data on sociodemographic and clinical variables, and other lung function risk factors, were collected using questionnaires: sex, age at baseline (i.e. ECRHS II), age completed full-time education (<17 years; 17–20 years; >20 years), occupation (management/professional/non-manual; technical/professional/non-manual; other non-manual; skilled manual; semiskilled/unskilled manual; other/unknown), childhood respiratory infection (yes/no) and occupational exposure to biological dust, gas/fumes or pesticides (yes/no). Number of pack-years smoked (calculated by multiplying the number of packs of cigarettes smoked per day by the number of years the person has smoked), second-hand smoke exposure (yes/no) and menopausal status in women (pre-menopausal/post-menopausal) were assessed at each survey. Dietary habits were collected by food frequency questionnaire once, for two centres at ECRHS II and 16 centres at ECRHS III, enabling the derivation of the alternative healthy eating index (AHEI-2010—a continuous measure of diet quality that is based on foods and nutrients predictive of chronic disease risk, range 0–110) [7] at either time-point. Height and weight (and hence body mass index (BMI)) were measured at each survey.

### Statistical analyses

**Fig 1:**
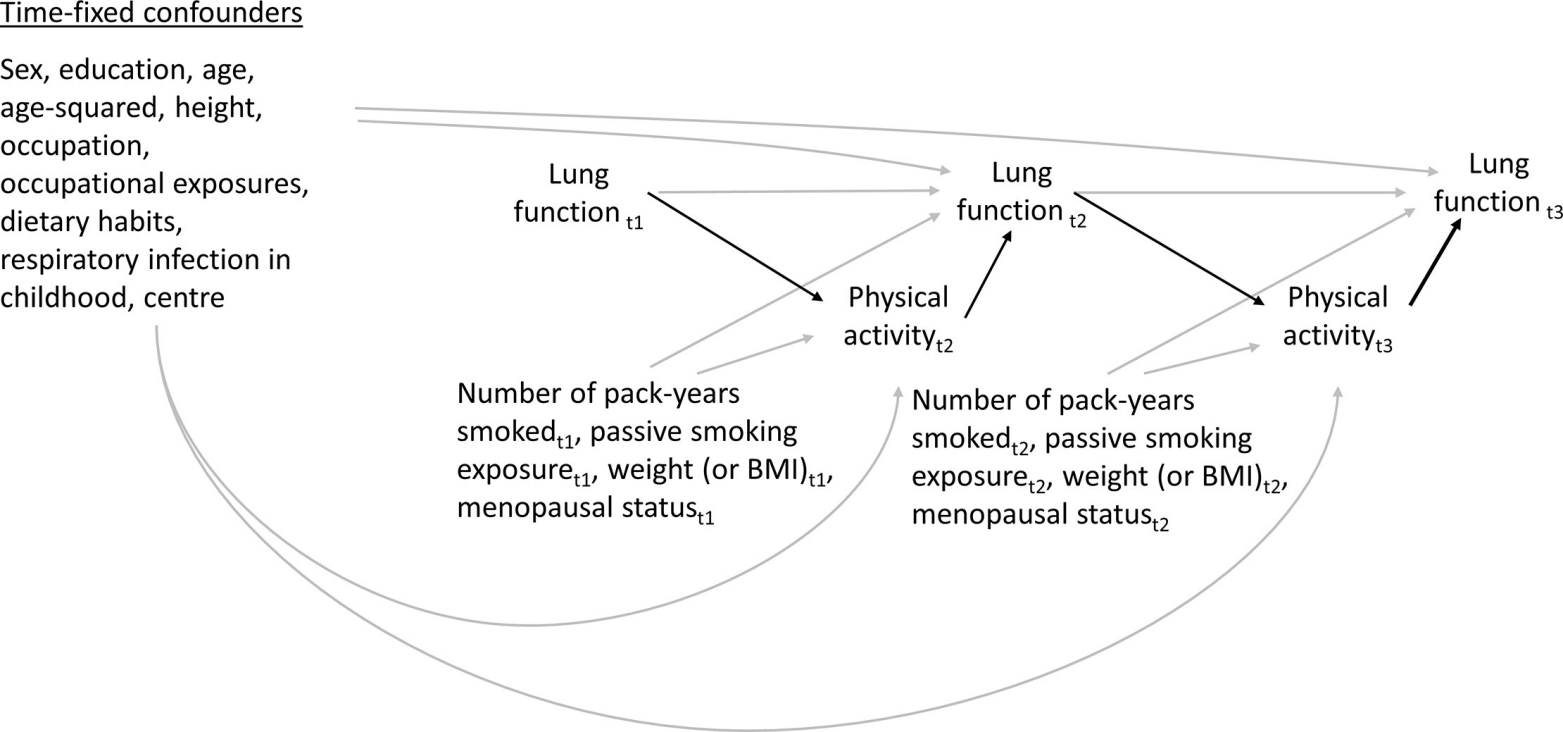
Directed acyclic graph showing potential time-fixed and time-dependent confounders of the association between physical activity and lung function over time in the ECRHS cohort.

Fig 1 depicts the hypothetical causal relationships tested in this study. Because physical activity was only assessed at ECRHS II and III, we considered, for both *t* = ECRHS II and III, the cross-sectional association between usual physical activity (i.e. the assessment of average physical activity overtime) at *t* and lung function at *t* as the causal effect of physical activity on lung function, and the association between lung function at *t*-1 and physical activity at *t* as the causal effect of lung function on physical activity.

The following variables were selected as time-fixed confounders in the analyses: sex, education, age, age-squared, height, occupation, the AHEI-2010 score, childhood respiratory infection and centre. The following variables were selected as time-dependent confounders in the analyses: number of pack-years smoked, second-hand smoke exposure and weight at *t*-1. As the inclusion of BMI and menopausal status may compromise statistical power because of their correlation with weight and age/age-squared, respectively, and the inclusion of occupational exposures compromised statistical power because of high missingness, the three variables were only considered as covariates in a sensitivity analysis.

We used generalized SEM (exploratory approach based on logistic and linear regression models) to test the existence of the hypothesised relationships (i.e. “paths”) depicted in Fig 1, and more particularly to investigate the bi-directionality of the association between physical activity and lung function, controlling for time-fixed and time-varying confounders [8]. The gsem command in STATA was used (more details are provided in the online S1 File). The association of physical activity with lung function was measured by the difference in expected lung function (β); the association of lung function with physical activity was measured by the odds ratio (OR).

We used MSM, an approach from the causal inference framework, to investigate whether the potential effect of physical activity on lung function remains after correcting for potential reverse causation (i.e. the potential effect of previous lung function on physical activity that may be suggested by the use of SEMs) and time-dependent confounding. MSMs were applied using inverse probability weighting, which inherently corrects for "cumulative confounding" throughout time, to allow the estimation of the *causal* effect of physical activity on lung function [9] by mimicking a hypothetical randomized experiment via the creation of a pseudo-population in which exposed and non-exposed subjects are exchangeable within levels of the available confounders [10] (more details, including STATA codes are provided in the online S1 File). The effect of physical activity on lung function was measured by the β coefficient.

As sensitivity analyses: (1) we used weight truncation (i.e. we reset the value of weights greater than the 95^th^ percentile to the 95^th^ percentile value and the value of weights lower than the 5^th^ percentile to the 5^th^ percentile value)., and (2) those who had avoided vigorous exercise because of wheezing or asthma at ECRHS II, as their inclusion may lead to an overestimation of the true protective effect of physical activity on lung function; (3) we repeated the MSMs analyses by restricting the study population to consistently active smokers throughout the follow-up (i.e. subjects who had reported current smoking at ECRHS I, II and III), and (4) by considering frequency of physical activity (≤once a month, 1–3 times per week and ≥4 times per week) and duration per week (≤30 minutes per week, 1–3 hours per week and ≥4 hours per week) as exposures of interest, in order to check the presence of a linear dose-response relationship between physical activity and lung function.

Analyses were conducted using STATA v14.0.

### Ethics statement

Ethical approval from the appropriate ethics committees was obtained by all centres participating in the ECRHS: Regional Committees for Medical and Health Research Ethics (REK Vest, number 2010/759, date: 22^nd^ march 2010) for Bergen (Norway); Ethik-Kommission der Bayerischen Landesärztekammer (number 10015, date: 8^th^ June 2010) for Hamburg and Erfurt (Germany); Comité Ético de Investigación Clínica del Instituto Municipal de Asistencia Sanitaria (number 2009/3500/l, date: 22^nd^ June 2010) for Barcelona, Galdakao, Albacete, Oviedo and Huelva (Spain); The National Bioethics Committee (NBCI, ref: VSNb2011090016/03.11, date: 30^th^ January 2015) for Reykjavik (Iceland); Regionala etikprövningsnämnden (number 2010/432, date: 12^th^ January 2011 and number 2010/068, date: 24^th^ March 2010) for Gothenburg, Umea and Uppsala (Sweden); Research Ethics Committee (REC, ref: 11/LO/0965, date: 7^th^ July 2011) for Ipswich and Norwich (UK); Comité de protection des personnes Sud Est V (ref: 11-CHUG-03, date: 3^rd^ March 2011) for Bordeaux, Grenoble, Montpellier and Paris (France). Written consent was obtained from all participants.

## Results

Table 1 shows the main characteristics of the 753 participants included in the study (mean age at ECRHS II: 41 years; female: 46%). Between 31% and 38% of these individuals were considered physically active over the study period. Compared to the subjects included in the study population, those excluded were more likely to be women and to report an unknown/other occupation, otherwise they were similar in terms of age, lung function, physical activity, smoking and other characteristics (S1 Table in online S1 File).

**Table 1:** Description of the study population (n = 753).

Time of assessment*	ECRHS I	ECRHS II (baseline)	ECRHS III
**Outcomes of interest (also considered as time-varying confounders)**			
**FEV**_**1**_ (mL), m (SD)	3.7 (0.8)	3.5 (0.7)	2.9 (0.7)
**FVC** (mL), m (SD)	4.5 (0.9)	4.3 (0.9)	4.0 (0.9)
**Exposure of interest**			
**Physical Activity**			
**Active (%)**	-	30.7	38.0
**Frequency (%)**			
≤1 a month		49.7	44.1
1–3 times a week	-	40.1	41.0
≥4 times a week		10.2	14.9
**Duration (%)**			
≤30 min		48.1	46.2
1–3 hours	-	39.0	34.1
≥4 hours		12.9	19.7
**Time-varying confounders**			
**Number of pack-years smoked, m (SD)**	13.1 (11.4)	21.5 (17.1)	
**Passive smoking (%)**	78.8	65.2	
**Weight** (kg), m (SD)	70.5 (13.3)	74.1 (14.7)	
**Menopausal status in women (%)**			
Pre-menopausal	96.1	84.2	
Post-menopausal	3.9	15.8	
**Time-fixed confounders**			
**Sex (%)**			
Female	45.5		
Male	54.5		
**Education (%)**			
<17 years	22.1		
17–20 years	34.6		
>20 years	43.3		
**Age** (years), m (SD)		41.4 (7.0)	
**Height** (cm), m (SD)	170.2 (8.9)	-	
**Occupation (%)**			
Management/professional/non-manual		26.6	
Technical/professional/non-manual		18.9	
Other non-manual		23.9	
Skilled manual		13.6	
Semiskilled/unskilled manual		13.0	
Other/unknown		4.1	
**Alternative healthy eating index-2010**^**±**^, m (SD)		50.4 (8.1)	50.4 (12.4)
**Respiratory infection during childhood (%)**	10.4		
**Occupational exposure to dust, gas/fumes or pesticides during follow-up (%)**		53.4	

m: mean; SD: standard deviation

*As shown in Fig 1, outcome data were considered at ECRHS I, II and III, exposure data were considered at ECRHS II and III, time-varying confounder data were considered at ECRHS I and II, and time-fixed confounder data were considered only once (i.e. when available).

^**±**^ The AHEI-2010 score was derived at ECRHS III for sixteen centres; two additional centres had dietary data at ECRHS II only.

**Fig 2:**
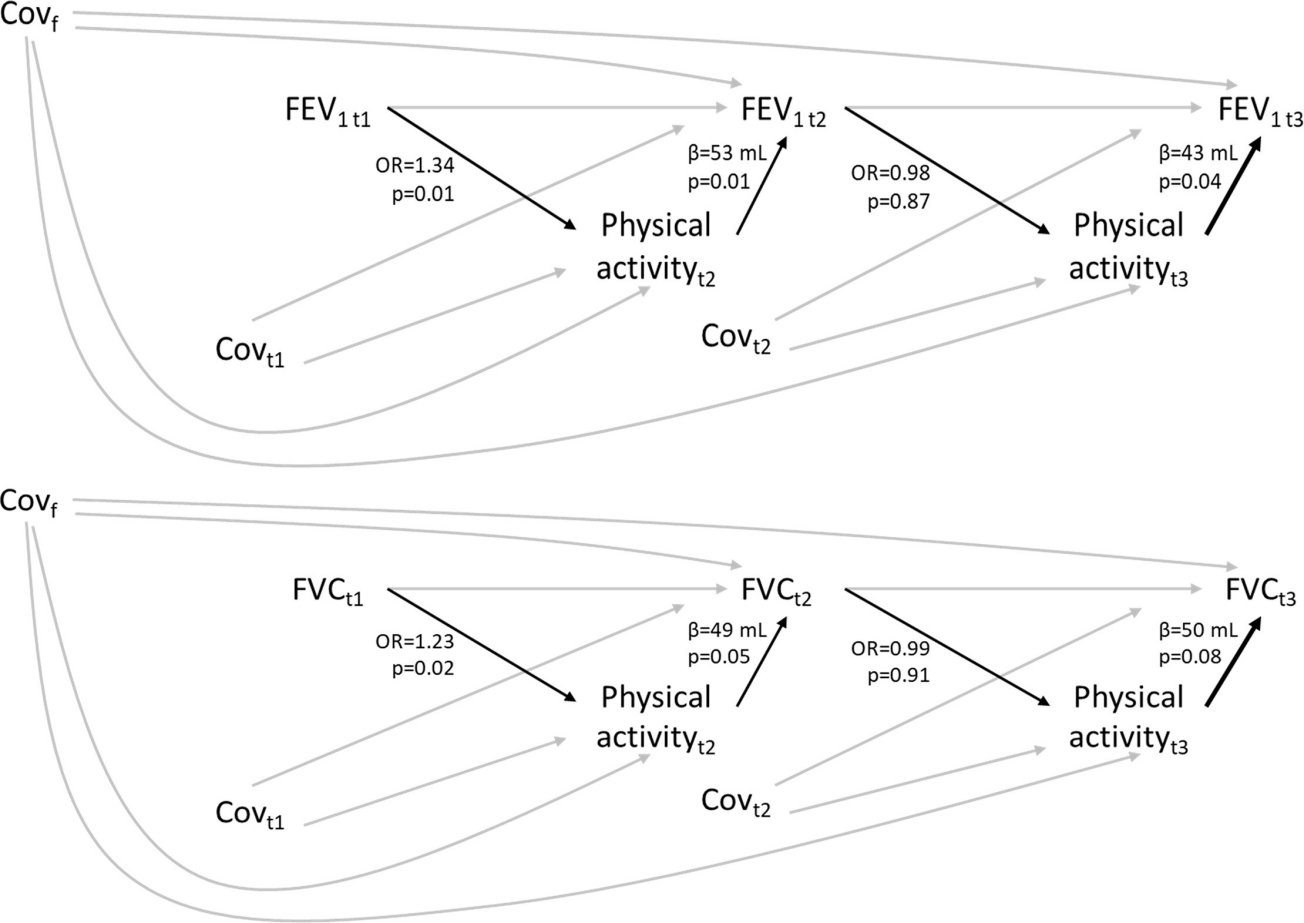
Associations of physical activity with lung function over time estimated using SEMs in the ECRHS cohort. Cov _t_ (time-varying confounders): number of pack-years smoked, passive smoking exposure, weight. Cov _f_ (time-fixed confounders): sex, education, age, age-squared, height, occupation, AHEI-2010 score, respiratory infection in childhood, centre. NB: The inclusion of BMI (instead of weight), menopausal status (in addition to age and age-squared), and occupational exposures compromised statistical power without substantially altering the results, thus they were not considered as covariates in the final models. β: difference in the expected lung function measure comparing active versus non-active individuals. OR: odds ratio comparing the risk of being active versus non-active for each 500 mL increase in lung function measures.

Using generalized SEMs, positive associations of physical activity on lung function parameters were found at both ECRHS II and III (difference in expected FEV_1_ (95%CI), active versus non-active: 53 mL (12, 94) and 43 mL (1, 85); difference in expected FVC (95%CI), active versus non-active: 49 mL (0, 98) and 50 mL (6, 106); see Fig 2). We only identified positive associations of lung function at ECRHS I on physical activity at ECRHS II (OR (95% CI), 500 mL increase in FEV_1_ 1.34 (1.09, 1.66); OR (95% CI), 500 mL increase in FVC: 1.23 (1.04, 1.46); see Fig 2).

The inclusion of BMI, menopausal status and occupational exposures as additional covariates did not substantially alter our results.

**Fig 3:**
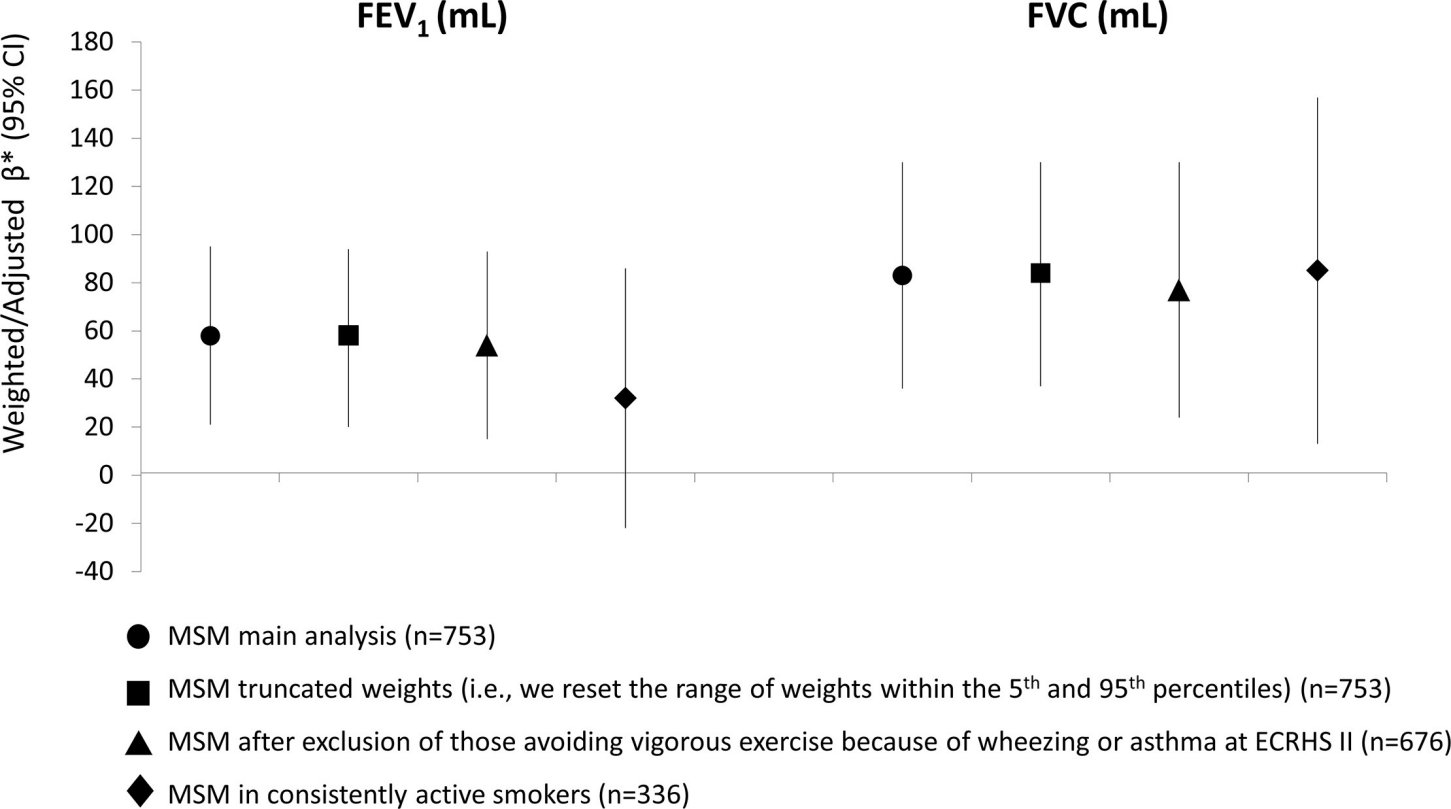
Effects of physical activity on lung function estimated using MSMs (main and sensitivity analysis) in the ECRHS cohort. β: difference in the expected lung function measure comparing active versus non-active individuals. *Models included. number of pack-years smoked, passive smoking exposure, weight, sex, education, age, age-squared, height, occupation, AHEI-2010 score, respiratory infection in childhood), and centre.

**Fig 4:**
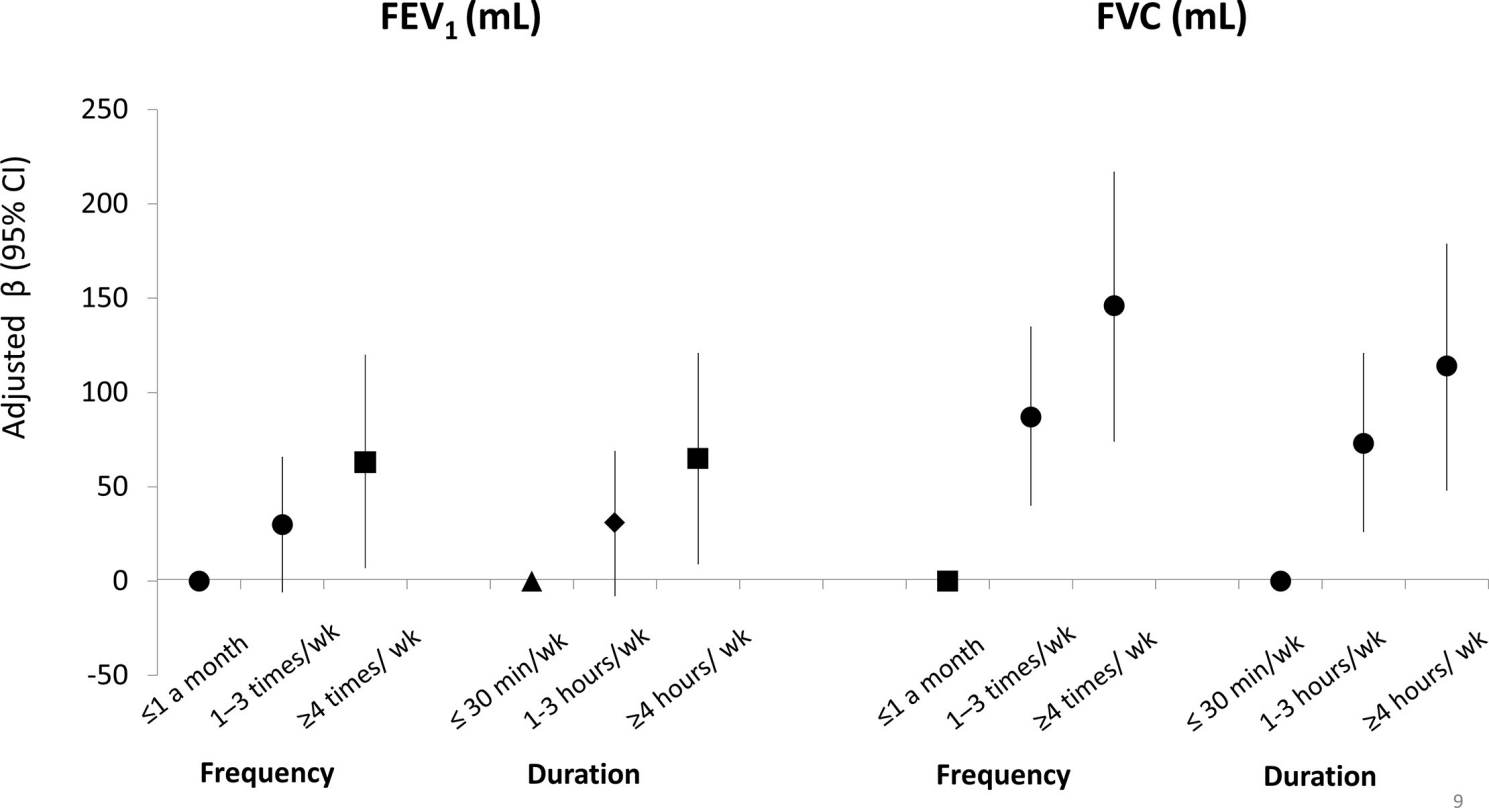
Effects of frequency and duration of physical activity on lung function, estimated using MSMs in the ECRHS cohort. β: difference in the expected lung function measure. *Models included number of pack-years smoked, passive smoking exposure, weight, sex, education, age, age-squared, height, occupation, AHEI-2010 score, respiratory infection in childhood), and centre.

Using MSMs, strong positive effects were found between being physically active and having higher lung function levels (difference in expected FEV_1_ (95% CI), active versus non-active: 58 mL (21–95); difference in expected FVC (95% CI), active versus non-active: 83 mL (36–130); see Fig 3). Similar effects were found when the MSM analyses were repeated using truncated weights, suggesting that the magnitude of time-dependent confounding is relatively low (Fig 3). When we repeated the MSM analysis including only the 336 subjects who had consistently reported being current smokers at ECRHS I, II and III, the estimated effects remained stable although results lost statistical significance (Fig 3). When the MSM analyses were conducted to investigate the effects of frequency and duration of physical activity on lung function, strong linear positive relationships were found (Fig 4).

## Discussion

This is the first longitudinal study among adult current smokers to investigate and report a positive bi-directional association between physical activity and lung function, although this finding was exploratory and not consistent throughout the study’s follow-up. The notion that lung function impacts physical activity likely comes from the fact that exercise limitation is a well-known consequence of respiratory conditions [11]. However, people with normal lung function (as is the case for most of our sample) have a wide range of ‘potential’ physical activity levels, and as physical activity is a behaviour, it is affected by many more factors other than lung function alone [12]. Thus, it is possible that the bi-directionality between physical activity and lung function can only be properly studied in other samples covering wider (i.e. including the lowest) ranges of both parameters, such as in clinical studies.

This study also found a positive effect between physical activity and lung function after removing potential time-dependent confounding and taking into account the association between previous lung function and physical activity.

Several studies have found positive associations between physical activity [1–3,13–21] and lung function levels in adults but most of them were cross sectional [15–17] or conducted in specific populations such as COPD patients [18] or adults with asthma [19]. A few prospective studies suggested a beneficial effect of physical activity on lung function in healthy adults [2,13,20,21] or in the general population [1,3,14], although results are inconsistent in terms of assessment of physical activity, length of follow-up or adjustment for potential confounders. The evidence linking regular physical activity and improved lung function is growing and appears to suggest stronger associations among current smokers [1,2]. Our results are consistent with the results from a previous MSM analysis conducted in the Copenhagen City Heart Study [3] and overcome some limitations by including dietary data. Our study also goes beyond that previous study by investigating more thoroughly reverse causation (i.e. studying also the potential effect of lung function on physical activity) and including a more geographically diverse population with a wider range of exposures, outcomes and covariates.

The use of MSMs and the fact that the results were robust to sensitivity analyses and are consistent with the literature [1,2] supports causal interpretation of the protective effect of physical activity on lung function.

A major strength of this study is the use of two complementary approaches to address a methodologically challenging research question. Other strengths include its longitudinal design, population-based nature, broad geographical representation of participants, and the availability of repeated measurements for outcome, exposure, and relevant confounders—some of which (e.g. diet) were not considered before.

This study’s main limitation is that the design of the ECRHS, with questionnaires administered ten-years apart, allowed only two cross-sectional estimations between physical activity and lung function, which may not allow time-dependent confounding to be fully addressed. However, it is worth mentioning that at the time of their lung function measurement, ECRHS subjects were asked about their *usual* physical activity. Hence assuming that physical activity at time t precedes lung function at time t seems reasonable. Moreover, as similar results were found after excluding those who had reported that they ‘avoided vigorous exercise because of wheezing/asthma’, suggesting that the positive effects found between physical activity and lung function are not driven by these subjects, residual time-dependent confounding is less likely to be an explanation. Another potential limitation is the information bias due to the misclassification of physical activity, although this potential error is likely to have been non-differential with respect to lung function, and thus would be expected to bias effect estimates towards the null. No information was available for *moderate* physical activity, which may be more beneficial for lung function [2], and no repeated information was available on body composition (only body weight). We cannot rule out the possibility that the exclusion of ECRHS participants without complete information for this specific analysis might have biased our findings. However, our analyses showed no relevant differences between the included and excluded subjects. Finally, although many known confounders were accounted for, we cannot rule out residual/unmeasured confounding, e.g. from socioeconomic status (adjusted for using years of education) or dietary habits (only assessed once).

In conclusion, our results suggest bi-directional causation and support a true protective effect of physical activity on lung function in smokers, after accounting for reverse causation and time-dependent confounding.

## Supporting information

S1 File (DOCX)
