# Supplementary material for: Physical activity and lung function—Cause or consequence?
Source: PLoS One. 2020 Aug 20;15(8):e0237769. doi: 10.1371/journal.pone.0237769 (PMC7446897; doi:10.1371/journal.pone.0237769)

**Online supplementary file**

**Physical activity and lung function - cause or consequence?**

Annabelle Bédard^1,2,3^, Anne-Elie Carsin^1,2,3^, Elaine Fuertes^1,2,3,4^, Simone Accordini^5^, Shyamali C Dharmage^6^, Vanessa Garcia-Larsen^7^, Joachim Heinrich^8,9^, Christer Janson^10^, Ane Johannessen^11,12^, Bénédicte Leynaert^13,14^, José Luis Sánchez-Ramos ^15^, Gabriela P Peralta^1,2,3^, Isabelle Pin^16,17,18^, Giulia Squillacioti^19^, Joost Weyler^20^, Deborah Jarvis^4,21^, Judith Garcia-Aymerich^1,2,3^

1. ISGlobal, Barcelona, Spain

2. Universitat Pompeu Fabra (UPF), Barcelona, Spain

3. CIBER Epidemiologia y Salud Pública (CIBERESP), Barcelona, Spain

4. National Heart and Lung Institute, Imperial College London, United Kingdom

5. Unit of Epidemiology and Medical Statistics, Department of Diagnostics and Public Health, University of Verona, Italy

6. Allergy and Lung Health Unit, School of Population and Global Health, University of Melbourne, Australia

7. Program in Human Nutrition, Department of International Health, Johns Hopkins Bloomberg School of Public Health, Baltimore, USA

8. Helmholtz Zentrum Munchen - German Research Center for Environmental Health, Institute of Epidemiology I, Munich, Germany

9. Institute and Outpatient Clinic for Occupational, Social and Environmental Medicine, University Hospital Munich, Ludwig Maximilians University Munich, Germany

10. Department of Medical Sciences: Respiratory, Allergy and Sleep Research, Uppsala University, Sweden

11. Centre for International Health, Department of Global Public Health and Primary Care, University of Bergen, Norway

12. Department of Occupational Medicine, Haukeland University Hospital, Bergen, Norway

13. Inserm, UMR 1152, Pathophysiology and Epidemiology of Respiratory Diseases, Paris, France

14. UMR 1152, University Paris Diderot, France

15. Pneumology Service, Juan Ramón Jiménez Hospital, Huelva, Spain

16. CHU Grenoble Alpes, Department of Pediatrics, Grenoble, France

17. INSERM, Institut for Advanced Biosciences, Grenoble, France

18. University Grenoble Alpes, France

19. Department of Public Health and Pediatrics, University of Turin, Italy

20. Department of Epidemiology and Social Medicine, University of Antwerp, Belgium

21. MRC-PHE Centre for Environment and Health, Imperial College London, UK

**Implementaton of SEMs**

SEMs were implemented using the gsem command of the STATA software, as follows:

*gsem (lf_t2 <- lf_t1 i.pa_t2 [list V] [list L_t1])*

*(pa_t2 <- lf_t1 [list V] [list L_t1], logit)*

*(lf_t3 <- lf_t2 i.pa_t3 [list V] [list L_t2])*

*(pa_t3 <- lf_t2 [list V] [list L_t2], logit)*

where:

- t1=ECRHS I, t2=ECRHS II and t3=ECRHS III

- pa_t is physical activity (binary in our example) at time t
- lf_t is lung function (i.e. the FEV_1_ or FVC measure in our example) at time t
- list_V is the list of time_fixed covariates: sex, education, age, age-squared, height, occupation, AHEI-2010 score, respiratory infection in childhood, and centre in our example (NB: the inclusion of occupational exposures compromised statistical power without substantially altering the results, thus it was not considered as covariate in the final models).
- list_L_t is the list of time-dependent covariates at time t: number of pack-years smoked, passive smoking exposure, weight in our example (NB: the inclusion of BMI (instead of weight) and menopausal status (in addition to age and age-squared) compromised statistical power without substantially altering the results, thus they were not considered as covariates in the final models).

The first line of the code models lung function at ECRHS II according to lung function at ECRHS I, physical activity at ECRHS II, time-fixed covariates and time-dependent covariates at ECRHS I, the second line models physical activity at ECRHS II according to lung function at ECRHS I, time-fixed covariates and time-dependent covariates at ECRHS I, the third line models lung function at ECRHS III according to lung function at ECRHS II, physical activity at ECRHS III, time-fixed covariates and time-dependent covariates at ECRHS II and the fourth line models physical activity at ECRHS III according to lung function at ECRHS II, time-fixed covariates and time-dependent covariates at ECRHS II.

**Implementation of MSMs**

Associations estimated in observational studies cannot usually be interpreted as causal effects, because the exposed and unexposed subjects are rarely exchangeable (i.e., exposed and unexposed subjects rarely share the same set of confounder values). MSMs address time-dependent confounding and, at the same time, allow estimation of causal effects in observational studies [1] by mimicking a hypothetical randomized experiment via creation of a pseudo-population in which exposed and non-exposed subjects are exchangeable within levels of the available confounders [2].

For that purpose, each subject will be assigned a weight proportional to the inverse of the probability that each subject had his own exposure (i.e. physical activity) history at a given time, given a chosen set of covariates [3]. Standardized weights for physical activity were stabilized and calculated as follows:

(a) *SW*(t) = $\prod_{s\leq t} \frac{P\left( E_{1}(s) \right| \bar{E_{1}} \left( s-1 \right), V)}{P\left( E_{1}(s) \right| \bar{E_{1}} \left( s-1 \right), \bar{L} \left( s-1 \right), V)}$^,^

where SW(t) represents the stabilized weight for physical activity at time t, E_1_ represents physical activity, L represents a vector of time-dependent covariates, including previous lung function, and V represents a vector of time-fixed covariates. The probabilities in the numerator and denominator were estimated through logistic regression models for the probability of being physically active at each time s.

The second step consists of estimating the causal effect of physical activity on lung function in the reweighted pseudo-population using a weighted pooled linear regression model (i.e. mixed model) for lung function for each subject at each time t, according to physical activity at time t. As the set of baseline covariates V was not adjusted for through the weights, these variables were added as covariates in the final model [4].

MSMs were implemented using the STATA software, considering a pooled dataset in which one observation corresponds to one subject at each time-point (i.e. two observations per subject) as follows:

We define:

- id is the unique identifier for each subject
- time (i.e. 1 or 2) is considered as a continuous covariate in our example (i.e. we assume a linear association between time and exposure)
- pa_t is physical activity (binary in our example) at time t
- pa_lag is physical activity (binary in our example) at time t-1 (i.e. previous examination)
- lf_t is lung function (i.e. the FEV_1_ or FVC measure in our example) at time t
- lf_lag is lung function (i.e. the FEV_1_ or FVC measure in our example) at time t-1 (i.e. previous examination)
- list_V is the list of time_fixed covariates: sex, education, age, age-squared, height, occupation, AHEI-2010 score, respiratory infection in childhood, and centre in our example (NB: the inclusion of occupational exposures compromised statistical power without substantially altering the results, thus it was not considered as covariate in the final models).
- list_L_lag is the list of time_dependent covariates at time t-1 (i.e. previous examination): lung function (i.e. lf_lag), number of pack-years smoked, passive smoking exposure, weight in our example (NB: the inclusion of BMI (instead of weight) and menopausal status (in addition to age and age-squared) compromised statistical power without substantially altering the results, thus they were not considered as covariates in the final models).

1) First step: weight calculation according to the stabilized weight equation (a) described above:

*/********** Numerator ***********/*

At each time t, according to the numerator of equation (a), we first model physical activity at time t according to physical activity at t-1 (only for t=2) and time-fixed covariates. Second, we estimate the probability of being physically active (using predict as a secondary step).

**For t=1 (ECRHS II)*

*xi:logistic pa_t [list V] time*

*predict numpa if e(sample)*

**For t=2 (ECRHS III)*

*xi:logistic pa_t i.pa_lag [list V] time*

*predict numpa if e(sample)*

We calculate the numerator as the product of all time-specific probabilities of being physically active up to that time:

*sort centre id time*

*by centre id: replace numpa=numpa*numpa[_n-1] if _n!=1*

*/********** Denominator ***********/*

At each time t, according to the denominator of equation (a), we first model physical activity at time t according to physical activity at t-1 (only for t=ECRHS II), time-fixed covariates and time-dependent covariates at time t-1. Second, we estimate the probability of being physically active (using predict as a secondary step).

**For t=1 (ECRHS II)*

*xi:logistic pa_t [list V] [list L_lag] time*

*predict denpa if e(sample)*

**For t=2 (ECRHS III)*

*xi:logistic pa_t i.pa_lag [list V] [list L_ lag] time*

*predict denpa if e(sample)*

We calculate the denominator as the product of all time-specific probabilities of being physically active up to that time:

*sort centre id time*

*by centre id: replace denpa=denpa*denpa[_n-1] if _n!=1*

We calculate a weight for each subject at each time period, defined as explained in the equation above (a) by dividing the numerator by the denominator:

*gen w=numpa/denpa*

2) Second step: reweighted pooled model

We apply the calculated weights at each time and for each subject in the final pooled reweighted model (linear mixed model using the option [pw=w]) for lung function according to physical activity, adjusting for time-fixed covariates, allowing to assess the association between physical activity and lung function correcting for time-dependent confounding:

*xi: mixed lf_t i.pa_t [list V] time [pw=w] || id :*

**References**

1. Robins JM, Hernán MA, Brumback B. Marginal structural models and causal inference in epidemiology. *Epidemiology* 2000; 11: 550–560.

2. Hernan M a. A definition of causal effect for epidemiological research. *J. Epidemiol. Community Heal.* 2004; 58: 265–271.

3. Moodie EEM, Stephens D a. Marginal Structural Models: unbiased estimation for longitudinal studies. *Int. J. Public Health* 2011; 56: 117–119.

4. Cole SR, Hernán M a. Constructing inverse probability weights for marginal structural models. *Am. J. Epidemiol.* 2008; 168: 656–664.

**Table S1.** Comparison between included and excluded subjects

|  | **Included (n=753)** | **Excluded (n=825)** | **P** |
| --- | --- | --- | --- |
| **FEV_1_** (mL), m (SD) | 3.5 (0.7) | 3.5 (0.7) | 0.32 |
| **FVC** (mL), m (SD) | 4.3 (0.9) | 4.4 (1.0) | 0.78 |
| **Physical Activity** |  |  |  |
| **Active (%)** | 30.7 | 28.9 | 0.45 |
| **Number of pack-years smoked, m (SD)** | 21.5 (17.1) | 22.6 (17.2) | 0.22 |
| **Passive smoking (%)** | 65.2 | 69.5 | 0.07 |
| **Weight** (kg), m (SD) | 74.1 (14.7) | 73.4 (15.3) | 0.29 |
| **Menopausal status in women (%)**  Pre-menopausal  Post-menopausal | 84.2  15.8 | 85.7  14.3 | 0.69 |
| **Sex (%)**  Female  Male | 45.5  54.5 | 54.1  45.9 | 0.001 |
| **Education (%)**  <17 years  17–20 years  >20 years | 22.1  34.6  43.3 | 22.3  38.8  38.9 | 0.16 |
| **Age** (years), m (SD) | 41.4 (7.0) | 41.8 (6.9) | 0.22 |
| **Height** (cm), m (SD) | 170.2 (8.9) | 169.9 (9.4) | 0.60 |
| **Occupation (%)**  Management/professional/non-manual  Technical/professional/non-manual  Other non-manual  Skilled manual  Semiskilled/unskilled manual  Other/unknown | 26.6  18.9  23.9  13.6  13.0  4.1 | 26.3  17.1  25.2  10.9  10.6  9.9 | <0.001 |
| **Alternative healthy eating index-2010**, m (SD) | 50.2 (12.1)* | 52.1 (12.5) | 0.03 |
| **Respiratory infection during childhood (%)** | 10.4 | 12.1 | 0.31 |
| **Occupational exposure to dust, gas/fumes or pesticides during follow-up (%)** | 53.4 | 50.4 | 0.24 |

m: mean; SD: standard deviation

**^*^** The AHEI-2010 score was only available for 267 out of the 825 excluded subjects (as having available AHEI-2010 was one of the selection criteria)

**Figure S1.** Flow-chart of the study population


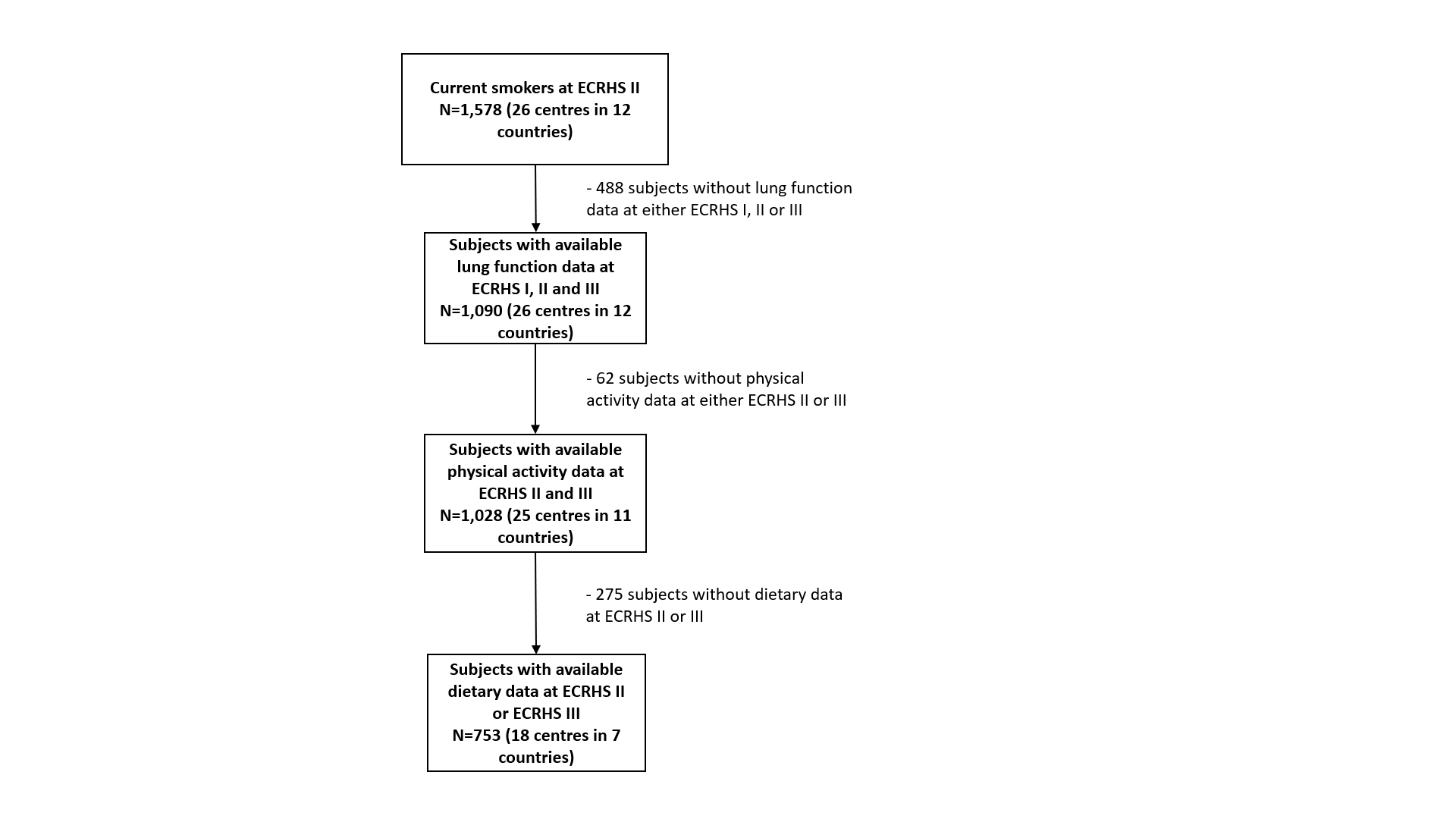

Supplement: S1 File — (DOCX) [file pone.0237769.s001.docx]
